# Tumor-Targeting Polymer–Drug Conjugate for Liver Cancer Treatment In Vitro

**DOI:** 10.3390/polym14214515

**Published:** 2022-10-25

**Authors:** Jiankun Xu, Shanmeng Lin, Hao Hu, Qi Xing, Jin Geng

**Affiliations:** 1 The First Clinical Medical College, Guangzhou University of Chinese Medicine, Guangzhou 510405, China; 2 Shenzhen Institute of Advanced Technology, Chinese Academy of Sciences, Shenzhen 518059, China

**Keywords:** polymer–drug conjugate, liver cancer, esterase responsive, bufalin

## Abstract

Bufalin (buf) has poor solubility in aqueous solution, poor tumor targeting, and many non-specific toxic and side effects. The advantages of high-molecular-weight polymer conjugates are that they can improve the water solubility of buf, prolong plasma half-life, and reduce non-specific toxicity. A novel water-soluble polymer–drug conjugate with buf and fluorescein pendants was prepared by the combination of reversible addition-fragmentation transfer (RAFT) polymerization and click chemistry. Its anticancer performance and cellular uptake behavior against liver cancer were investigated in vitro. The polymer–buf conjugates exhibit controlled release and tumor-targeting capabilities, showing promise for clinical applications.

## 1. Introduction

Liver cancer ranks in the top three in mortality rate among all tumors and is the sixth most common cancer worldwide in 2020 [1,2]. The disease is one of the worst cancers with a poor prognosis and a 5 year survival rate of less than 10% [3,4]. Treatment strategies for liver cancer include surgery, transplantation, transcatheter arterial chemoembolization (TACE), local ablation, chemotherapy, targeted therapy, immunotherapy, and traditional Chinese medicine (TCM) [5]. TCM plays an indispensable role as a complementary and alternative therapy for end-stage liver disease, such as cirrhosis or liver cancer [6,7]. Compared with synthetic drugs, TCM therapy, which is natural product, has the advantages of being less costly and having fewer adverse reactions for the treatment of liver cancer, improving survival and clinical benefit in patients [8,9]. Bufalin (buf), a toxic ligand and active compound, is extracted from toad venom [10]. Micromolar doses of buf can effectively kill human liver cancer cells [11,12]. However, buf has poor solubility in aqueous solution, poor tumor targeting, many non-specific toxic and side effects, easy decomposition after oral administration, a short half-life and low overall anticancer efficiency [13,14]. Suspending agents or auxiliary solvent can help disperse buf into aqueous solutions, but can also cause associated toxicity [15].

Water-soluble polymer–drug conjugates with targeting moieties have good biocompatibility, can prolong the residence time in blood circulation, actively deliver in cells, effectively accumulate in tumor sites, and improve pharmaceutical efficiency [16,17,18,19]. The polymer–drug conjugates with active organ-targeting properties can minimize the drug interaction with non-target organs to attenuate the side effects and toxicity. There are specific receptors on the surface of hepatoma cells that have an affinity for specific ligands [20]. Wu et al. synthesized galactosylated and fluorescein isothiocyanate-labeled polycaprolactone-g-dextran (Gal-PCL-g-Dex-FITC) polymers which could be selectively recognized by HepG2 cells and subsequently accumulate in HepG2 cells [21]. Ma et al. reported that because SMMC7221 human liver cancer cells overexpress lactose or galactose receptors, lactose-containing copolymers could be specifically and effectively internalized by SMMC7221 cells [22]. Xu et al. developed galactosamine-decorated PEGylated hyaluronan copolymers which could be efficiently internalized by HepG2 cells [23]. The receptors on the surface of hepatoma cells could be recognized by sugar ligands so that the above mentioned polymer–drug conjugates may be used as drug-vehicles for hepatoma-cell targeting drug delivery. Covalently linking drugs to polymer backbone is possible to control the cleavage and release of cytotoxic agents to avoid premature/burst drug release [24,25,26]. It was reported that polymer–drug conjugates can improve the aqueous solubility of buf, extend plasma half-life, and reduce non-specific toxicity [27].

The purpose of this study was to develop a mannose-modified polymer-drug conjugate for targeted intracellular delivery of buf in hepatoma cells in vitro. We combined the advantages of polymer–drug conjugates and ligand–receptor targeting strategies to achieve multiple functions of polymers, including sustained drug release, biodegradation, targeting of liver cancer cells, rapid cellular uptake, and fluorescence detection. The polymer–drug conjugates were synthesized by reversible addition-fragmentation transfer (RAFT) polymerization followed by click (alkyne-azide) reactions. In particular, we used aryl mannose residues as both water-soluble agents and targeting moieties. As we reported previously, the introduction of mannose groups can enhance cellular uptake in human hepatoma (HepG2) cells [28]. NMR was performed to characterize the polymer–buf conjugates. The anticancer performance of polymer–buf conjugates against HepG2 was evaluated. Flow cytometry and confocal laser scanning microscopy (CLSM) were conducted to examine the cellular uptake behavior of polymer–buf conjugates.

## 2. Materials and Methods

### 2.1. Materials

2,2′-Azobis(2-methylpropionitrile) (AIBN; Aladdin) was recrystallized from 95% ethanol. Buf (99%) was purchased from Chengdu Aifa Biotech Co., Ltd. (Chengdu, China) and used as received. Et_3_N, 2-Bromoethanol, 4-dimethylaminopyridine (DMAP), CuSO_4_·5H_2_O, Vitamine C sodium salt and Pent-4-ynoic acid were purchased from Energy Chemical Co., Ltd. (Shanghai, China) and used as received. D-(+)-Mannose was purchased from Aladdin Co., Ltd. (Shanghai, China) and used as received. KN_3_ and acryloyl chloride were purchased from Macklin Biochemical Co., Ltd. (Shanghai, China) and used as received. N,N′-dicyclohexylcarbodiimide (DCC) and 2,2′-azobis [2-(2-imidazolin-2-yl)propane]dihydrochloride (VA-044) were purchased from Bidepharm Co., Ltd. (Shanghai, China) and used as received. Propargylamine was purchased from Tokyo Chemical Industry Co., Ltd. (Shanghai, China) and used as received. LysoTracker^®^Red DND-99, Fetal bovine serum (FBS), penicillin, streptomycin, and Dulbecco’s Modified Eagle’s Medium (DMEM) were purchased from Thermo Fisher Scientific (Waltham, MA, USA) and used as received. Hoechst 33342 was purchased from Maokang Biotechnology Co., Ltd. (Shanghai, China) and used as received. The human liver cancer cell line HepG2 was purchased from the Cell Bank of Type Culture Collection of Chinese Academy of Sciences (Shanghai, China).

### 2.2. Compounds Synthesis

**Scheme 1:**
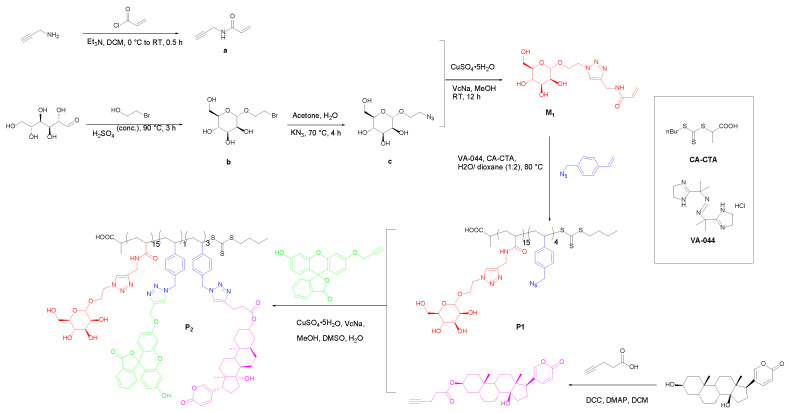
The flowchart of the synthesis process.

**Scheme 2:**
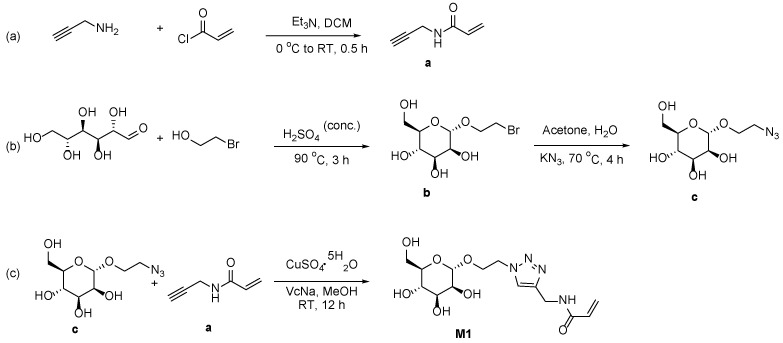
Synthesis of mannose acrylamide M1.

**Scheme 3:**
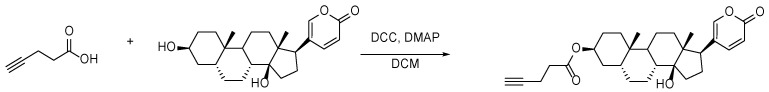
Synthesis of bufalin ester.

**Scheme 4:**
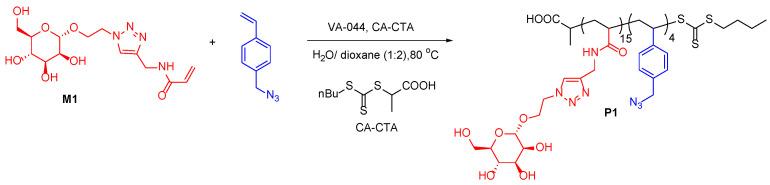
Synthesis of copolymer P1.

**Scheme 5:**
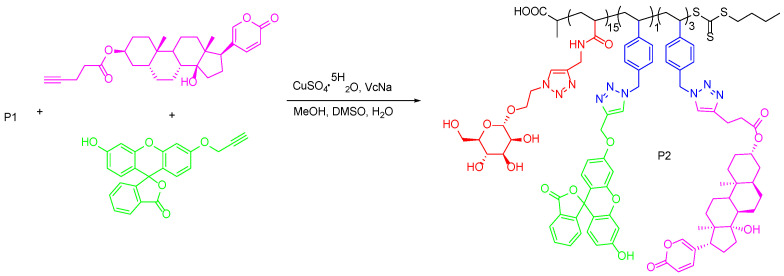
Synthesis of polymer–buf conjugate P2.

The flowchart of the complete synthesis process is shown in Scheme 1. The synthetic route for the preparation of mannose acrylamide M1 is shown in Scheme 2. The synthetic route for the preparation of bufalin ester is shown in Scheme 3. The synthetic route for the preparation of the copolymer P1 is shown in Scheme 4. The synthetic route for the preparation of the polymer–buf conjugate P2 is shown in Scheme 5.

#### 2.2.1. Synthesis of a

Propargylamine (2.20 g, 0.04 mol) and Et_3_N (6.07 g, 0.06 mol) were dissolved in 20 mL of CH_2_Cl_2_. The reaction mixture was cooled to 0 °C in an ice bath. Then, acryloyl chloride (3.62 g, 0.04 mol) was added dropwise. After the reaction, 100 mL of CH_2_Cl_2_ and 30 mL of water were used for extraction, and the aqueous phase was added, which was 30 mL of CH_2_Cl_2_. The obtained organic phases were dried with anhydrous Na_2_SO_4_ under reduced pressure at room temperature. The crude product was purified by column chromatography using petroleum ether/ethyl acetate (2:1 *v/v*) as the eluent to obtain a (2.36 g, yield 54.06%). ^1^H NMR characterization result of a is shown in Figure S1.

#### 2.2.2. Synthesis of b

D-(+)-Mannose (5.04 g, 28 mmol) and 2-bromoethanol (17.51 g, 0.14 mol) were charged into a 250 mL flask equipped with a magnetic stirring bar. Silica gel powder (3 g) and concentrated sulfuric acid (1 mL) were added to the flask. The reaction mixture was thermostatted at 90 °C in an oil bath and stirred for 3 h after connecting the condenser tube. The mixture was purified by column chromatography using CH_2_Cl_2_/MeOH (10:1 *v/v*) as the eluent to obtain b (5.42 g, yield 67.42%). The ^1^H NMR characterization result of b is shown in Figure S2.

#### 2.2.3. Synthesis of c

The compounds b (5.42 g, 18.88 mmol) and KN_3_ (1.85 g, 22.81 mmol) were dissolved in H_2_O/Acetone (1/1, 8 mL). The reaction mixture was thermostatted at 70 °C in an oil bath and stirred for 4 h after connecting the condenser tube. The mixture was purified by silica gel column chromatography using CH_2_Cl_2_/MeOH (10:1 *v/v*) as the eluent to obtain c (2.11 g, yield 44.84%). ^1^H NMR characterization result of c is shown in Figure S3.

#### 2.2.4. Synthesis of Mannose Acrylamide M1

The compounds a (1.11 g, 10.16 mmol) and c (2.11 g, 8.47 mmol) were dissolved in 8 mL of methyl alcohol. Then, CuSO_4_·5H_2_O (104.87 mg, 0.42 mmol), and Vitamine C sodium salt (166.41 mg, 0.84 mmol) were added. The reaction mixture was stirred at room temperature for 12 h. The mixture was purified by silica gel column chromatography using CH_2_Cl_2_/MeOH (5:1 *v/v*) as the eluent to obtain mannose acrylamide M1 (2.21 g, yield 72.81%). ^1^H NMR characterization result of mannose acrylamide M1 is shown in Figure S4.

#### 2.2.5. Synthesis of Buf Ester

Buf (77.3 mg, 0.20 mmol), pent-4-ynoic acid (23.52 mg, 0.24 mmol), DCC (51.58 mg, 0.25 mmol), and DMAP (2.44 mg, 0.02 mmol) were dissolved in 2 mL of CH_2_Cl_2_ and stirred at room temperature for 24 h. The mixture was purified by silica gel column chromatography using CH_2_Cl_2_/petroleum ether (3:1 *v/v*) as the eluent to obtain buf ester as a white solid (60.10 mg, yield 64.40%). ^1^H NMR characterization result of buf ester is shown in Figure S5.

#### 2.2.6. Synthesis of Copolymer P1

RAFT polymerization was used to synthesize the azide-terminated copolymers. In summary, 1-(azidomethyl)-4-vinylbenzene (7.96 mg, 0.05 mmol), CA-CTA (1.19 mg, 5.00 μmol), mannose acrylamide M1 (35.84 mg, 0.10 mmol), and 2,2′-azobis [2-(2-imidazolin-2-yl)propane]dihydrochloride (VA-044) (0.52 mg, 1.61 μmol) were dissolved in H_2_O/1,4-dioxane (1/2, 100 μL). The polymerization solution was bubbled with N_2_ for 30 min, then flame-sealed and polymerized at 80 °C for 24 h. After polymerization, the mixture was purified by dialysis (cellulose membrane; molecular weight cutoff (MWCO) 1000 Da) in deionized water for 48 h and lyophilized for a yield of 22.17 mg. The conversions of 1-(azidomethyl)-4-vinylbenzene and mannose acrylamide M1 were determined by ^1^H NMR to be ~40% and ~75%, respectively. The degree of copolymer was determined to be ~63.3 by ^1^H NMR analysis in DMSO. The ^1^H NMR characterization result of copolymer P1 is shown in Figure S6. The sample pictures of copolymer P1 are shown in Figure S7.

#### 2.2.7. Binding of Fluorescein Molecule and Buf

The copolymer P1 (80.68 mg), buf ester (18.17 mg, 0.04 mmol), and propargylic florescein (4.82 mg, 0.01 mmol) were dissolved in H_2_O/MeOH (1/1, 150 μL); 100 μL of DMSO was added. Then, CuSO_4_·5H_2_O (0.32 mg, 1.30 μmol) and Vitamine C sodium salt (0.52 mg, 2.62 μmol) were added. The reaction mixture was stirred at room temperature overnight. After the reaction, a small amount of CH_2_Cl_2_ and a large amount of water were used for extraction, and the aqueous phase was lyophilized to obtain the polymer–buf conjugate P2 (yield 49.96 mg). The sample pictures of polymer-buf conjugate P2 are shown in Figure S8.

### 2.3. Cell Culture

The human liver cancer cell line HepG2 cells were cultured in DMEM supplemented with 10% FBS, penicillin, and streptomycin under a humidified atmosphere of 5% CO_2_ at 37 °C.

### 2.4. Cell Viability Assays

HepG2 cells were seeded into a 96-well plate (0.8 × 10^4^ cells/well) and cultured overnight. The polymer–buf conjugate P2 (0.98 mg/mL) in DMEM was added and cultured for 48 h. The media was removed and incubated with 10 µL CCK-8 solution in 100 µL DMEM at 37 °C for 0.5 h. The absorbance at 450 nm was measured on a multimode plate reader and cell viability was calculated compared to untreated cells. The cytotoxicity of free buf and the copolymer was examined by the same method.

### 2.5. Cellular Uptake Behavior of Polymer-Buf Conjugate P2 by Flow Cytometry

HepG2 cells (3 × 10^5^ per well) were seeded into six-well cell culture plates and cultured for 24 h. Next, the original medium was replaced with a fresh medium containing polymer–buf conjugate P2 (98 μg/mL) for 2, 4, 5 h. Afterwards, the culture medium was removed, and cells were washed with PBS for 3 times and harvested with trypsin. The cells were resuspended in 300 µL of PBS. The targeting efficiency of the polymer–buf conjugate P2 in vitro was assessed using flow cytometry (Calibur; BD Biosciences, Franklin Lakes, NJ, USA).

### 2.6. Fluorescence Imaging

HepG2 cells were seeded into CLSM-specific dishes at a density of 3 × 10^5^ cells per dish and cultured overnight. Subsequently, the cells were cultured with fresh medium containing polymer–buf conjugate P2 at a buf concentration of 1.4 μg/mL for 2, 4, 5 h. Afterward, the culture medium was removed. The cells were washed with PBS 3 times, and stained with LysoTracker^®^Red DND-99 and Hoechst 33342 for 30 min and 15 min, respectively. Next, the cells were washed with PBS 3 times and cultured with PBS. The samples were visualized using CLSM (Leica Microsystems, Mannheim, Germany). λ_ex/em_ (LysoTracker^®^Red DND-99) = 577/590 nm, λ_ex/em_ (copolymer–buf conjugate) = 498/517 nm, λ_ex/em_ (Hoechst 33342) = 346/460 nm.

### 2.7. Statistical Analysis

All statistical analyses and graphs were generated with GraphPad Prism 8.0 (GraphPad Software, Inc., La Jolla, CA, USA). Each experiment was performed in triplets and the quantitative data are presented as mean ± standard deviation.

## 3. Results and Discussion

### 3.1. Synthesis and Characterization of Polymer-Buf Conjugate

**Figure 1:**
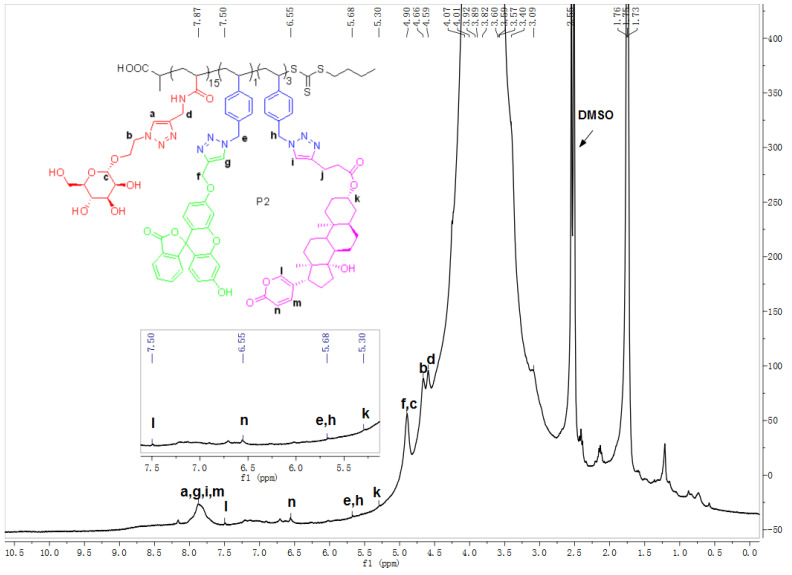
^1^H spectrum of polymer–buf conjugate P2 recorded in d_6_-DMSO.

Polymer–drug conjugates containing mannose as a targeting and water-soluble agent, buf as a drug candidate and fluorescein as a fluorescence agent was prepared by RAFT polymerization and click chemistry. A random copolymer P1 was first prepared by RAFT polymerization of mannose monomer (M1) and 1-(azidomethyl)-4-vinylbenzene. The fluorescent agents and the drug molecules (buf) were introduced and formed the polymer–drug conjugate P2 through CuAAC click reaction. ^1^H NMR analysis further confirmed the successful linkage of buf and the fluorescein molecule onto the polymer backbone (Figure 1).

### 3.2. In Vitro Cellular Uptake of Polymer-Buf Conjugate P2

**Figure 2:**
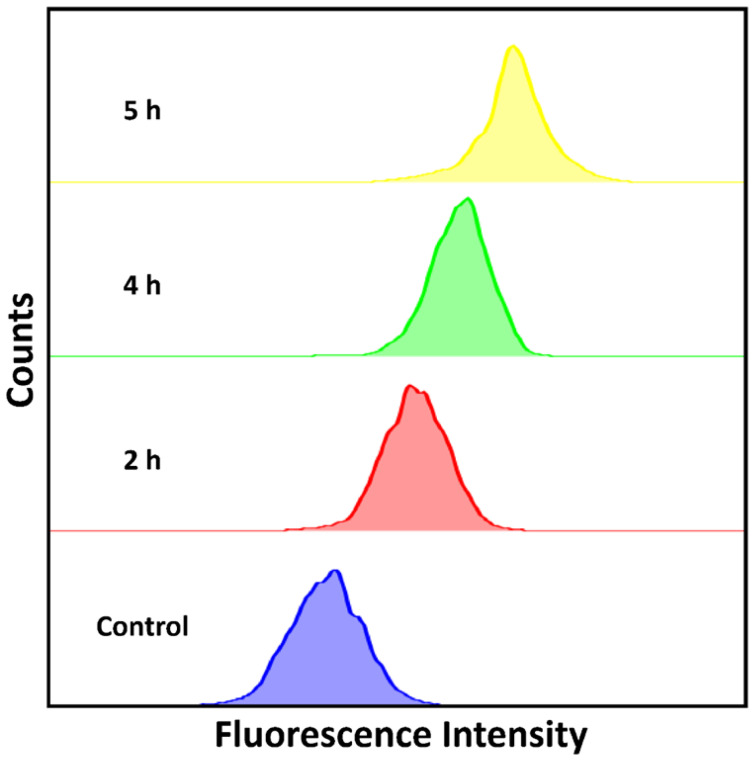
Flow cytometry analysis of cellular uptake of the polymer–bufalin conjugate P2 for 2, 4, and 5 h in HepG2 cells.

**Figure 3:**
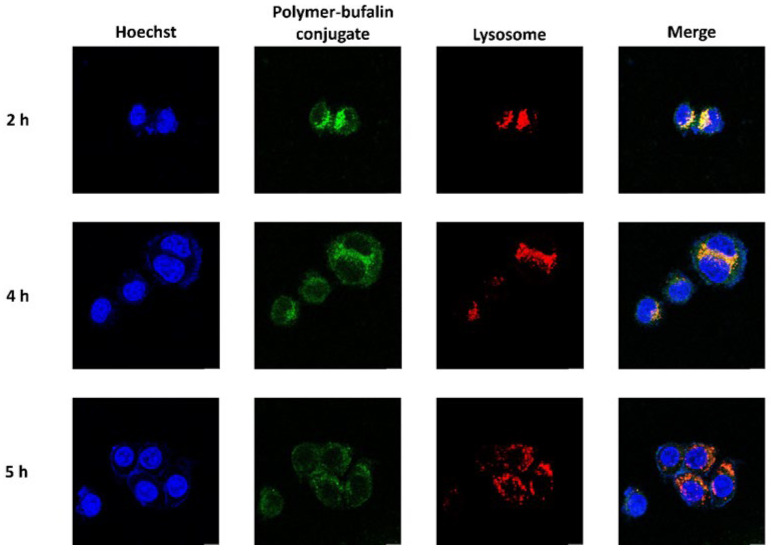
Visualization of cellular uptake of the polymer–bufalin conjugate P2. HepG2 cells were treated with polymer–buf conjugate P2 for 2, 4, and 5 h and analyzed by the confocal microscope. Scale bar = 100 μm.

Tumor-targeting polymer–drug conjugates can selectively deliver drugs to tumor cells to achieve better therapeutic effects [29]. The flow cytometry was first used to quantitatively evaluate the selective cellular uptake mechanism of the polymer–buf conjugate P2 in HepG2 cells. Compared with the control sample, the polymer–buf conjugate P2 showed stronger fluorescence emission with the prolongation of incubation time from 2 to 5 h, indicating that the polymer–buf conjugate P2 can effectively enter into cells and improve the internalization of drugs (Figure 2). The intracellular distribution of the polymer–buf conjugate P2 was further investigated using a microscope. The cell nuclei were stained with Hoechst 33342, appearing blue; the lysosome was stained with LysoTracker^®^Red DND-99 as red; the green color represents the polymer–buf conjugate P2 in cells. When the cells were incubated with polymer–buf conjugate P2 in 2 h, the green signals of the polymer molecules were mostly observed in the lysosome, suggesting the preferential accumulation of the polymer–buf conjugate P2 in the lysosome. Subsequently, the polymer–buf conjugate P2 diffused to the cytosol over the time (5 h) as confirmed by green fluorescence change. (Figure 3). The complementarity of the two methods, quantitative flow cytometry and qualitative fluorescence, proved to be a useful tool for the study of the cellular uptake of polymer–drug conjugates. Fluorescence properties can be used to track therapeutic molecule delivery, allowing for the evaluation of cell-based therapy. Furthermore, adding fluorescence properties to these polymer–drug conjugates offers new potential for in vitro direct imaging and localization in living cells. Our current study with fluorescent polymer–drug conjugates allows for the visualization of their interactions with HepG2 cells.

### 3.3. In Vitro Cytotoxicity

**Figure 4:**
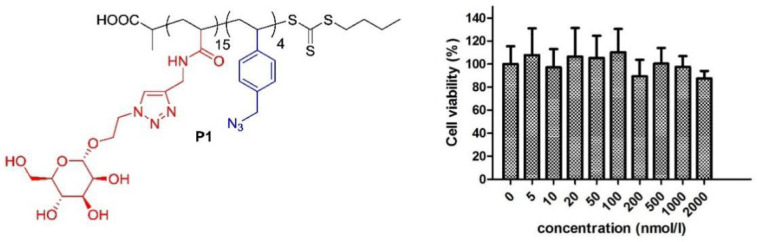
HepG2 cell viability against copolymer P1 (CCK-8 assay, 48 h).

**Figure 5:**
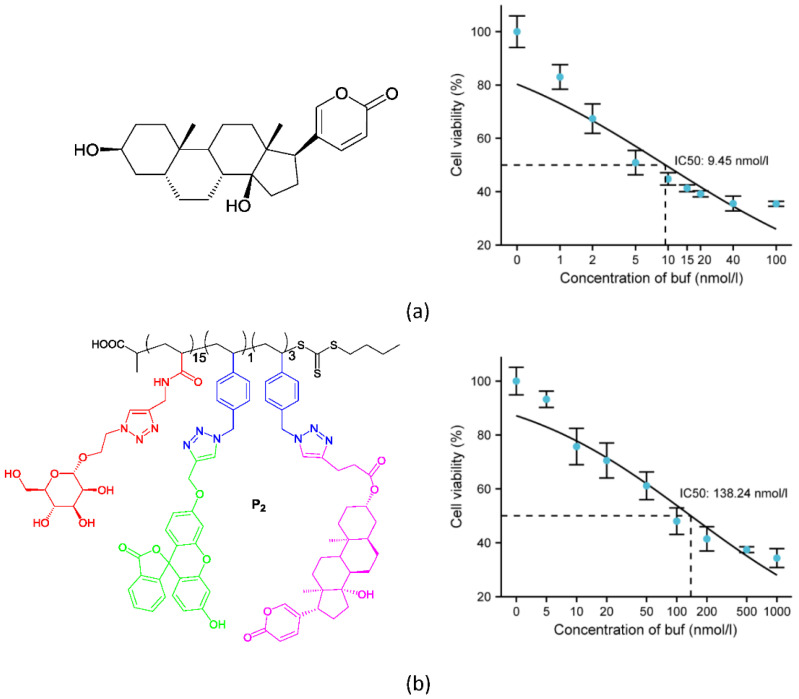
HepG2 cell viability against (**a**) free bufalin and (**b**) the polymer–bufalin conjugate P2 at different concentration of buf (CCK-8 assay, 48 h).

The anticancer performance of the synthesized polymer–buf conjugate P2 was evaluated by human liver cancer cell line HepG2 cells. The copolymer P1 was first examined. All of the concentrations of the copolymer P1 exhibited low cytotoxicity towards HepG2 cells. The cell viability was over 80% even at a high copolymer concentrations (2 μmol L^−1^), indicating that the copolymer P1 is highly biocompatible and nontoxic (Figure 4). Thus, the copolymer P1 designed in this study can be a promising candidate for polymer–drug conjugation. Furthermore, we measured the half maximal inhibitory concentration (IC50) of buf as 9.45 nm/L. When compared with free buf, the cytotoxicity induced by the treatment of polymer–buf conjugate P2 was significantly attenuated. The IC50 in the polymer–buf conjugate P2 treatment group was found to be 138.24 nm/L (with respect to buf units, Figure 5). The polymer–buf conjugate P2 contains ester bonds that can be specifically hydrolyzed by esterase. Due to the response of the polymer–buf conjugate P2 to esterase, the polymer–drug conjugate reported in this study reduced the toxicity of buf in tumor cells, providing further proof that the esterase-responsiveness of the polymers ensured an efficient degradation of linkers between the buf and polymer backbone with a sustained release of buf.

The results of cell uptake and cytotoxicity in vitro suggested that the polymer–buf conjugate P2 could be effectively taken up and activated by esterase-positive cancer cells, thus releasing cytotoxic drugs.

## 4. Conclusions

In summary, we have successfully constructed novel water-soluble polymer–drug conjugates based on mannose-targeting moieties and hydrolyses of ester bonds. Compared to the common free drug bufaline, the prepared polymer–buf conjugates exhibited lower non-specific toxicity and tumor uptake, showing controlled release for potential clinical applications.

## Data Availability

All data is available in supporting information.

## Supplementary Materials

The following supporting information can be downloaded at: https://www.mdpi.com/article/10.3390/polym14214515/s1, Figure S1: ^1^H NMR characterization result of a; Figure S2: ^1^H NMR characterization result of b; Figure S3: ^1^H NMR characterization result of c; Figure S4: ^1^H NMR characterization result of mannose acrylamide M1; Figure S5: ^1^H NMR characterization result of buf ester; Figure S6: ^1^H NMR characterization result of copolymer P1. Figure S7: The sample picture of copolymer P1. Figure S8: The sample picture of polymer-buf conjugate P2.

## Abbreviations

LIHC: liver hepatocellular carcinoma; Buf: bufalin; RAFT: reversible addition-fragmentation transfer; TACE: transcatheter arterial chemoembolization; TCM: traditional Chinese medicine; CLSM: confocal laser scanning microscopy.
