# Supplementary material for: Tumor-Targeting Polymer–Drug Conjugate for Liver Cancer Treatment In Vitro
Source: Polymers (Basel). 2022 Oct 25;14(21):4515. doi: 10.3390/polym14214515 (PMC9653589; doi:10.3390/polym14214515)
Supplement: Supplementary file 1 [file polymers-14-04515-s001.zip › polymers-1956848-supplementary.pdf]

Supplementary Information

Tumor-Targeting Polymer-Drug Conjugate for Liver Cancer Treatment in vitro

Jiankun Xu[1,2], Shanmeng Lin[2], Hao Hu[1*], Qi Xing[2*], Jin Geng[2*]

[1] The First Clinical Medical College of Guangzhou University of Chinese Medicine, Guangzhou 510405, China

[2] Shenzhen Institute of Advanced Technology, Chinese Academy of Sciences, Shenzhen 518059, China

* Correspondence: huhao_hbtcm@163.com (H.H.); q.xing@siat.ac.cn (Q.X.); jin.geng@siat.ac.cn (J.G.)

Supporting Figures

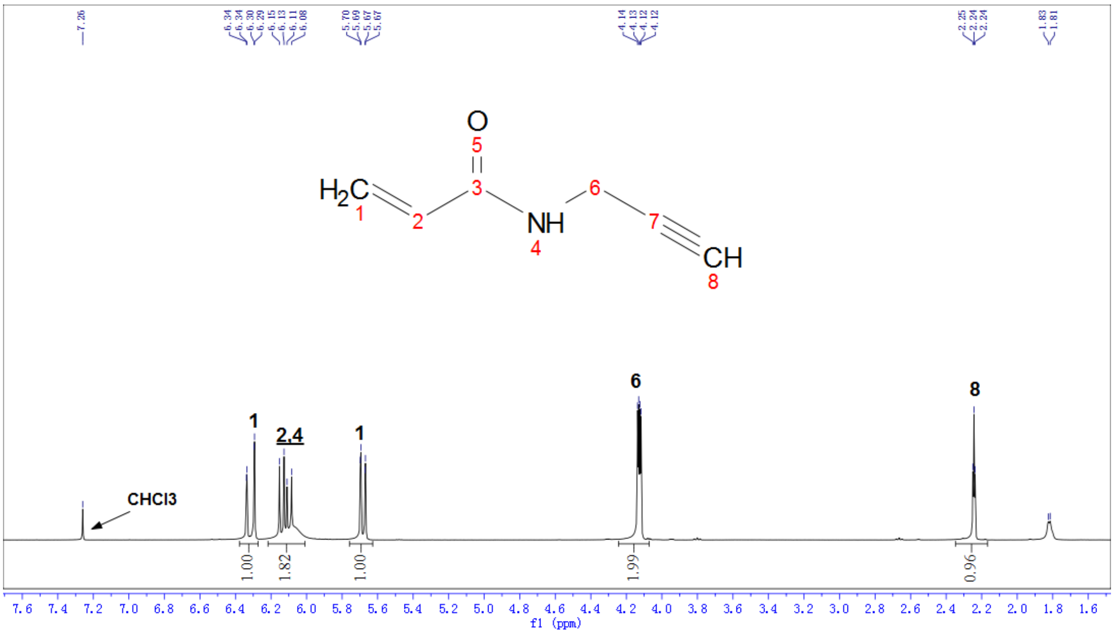

Figure S1. ^{1}H spectrum of **a** recorded in $CDCl_3$.

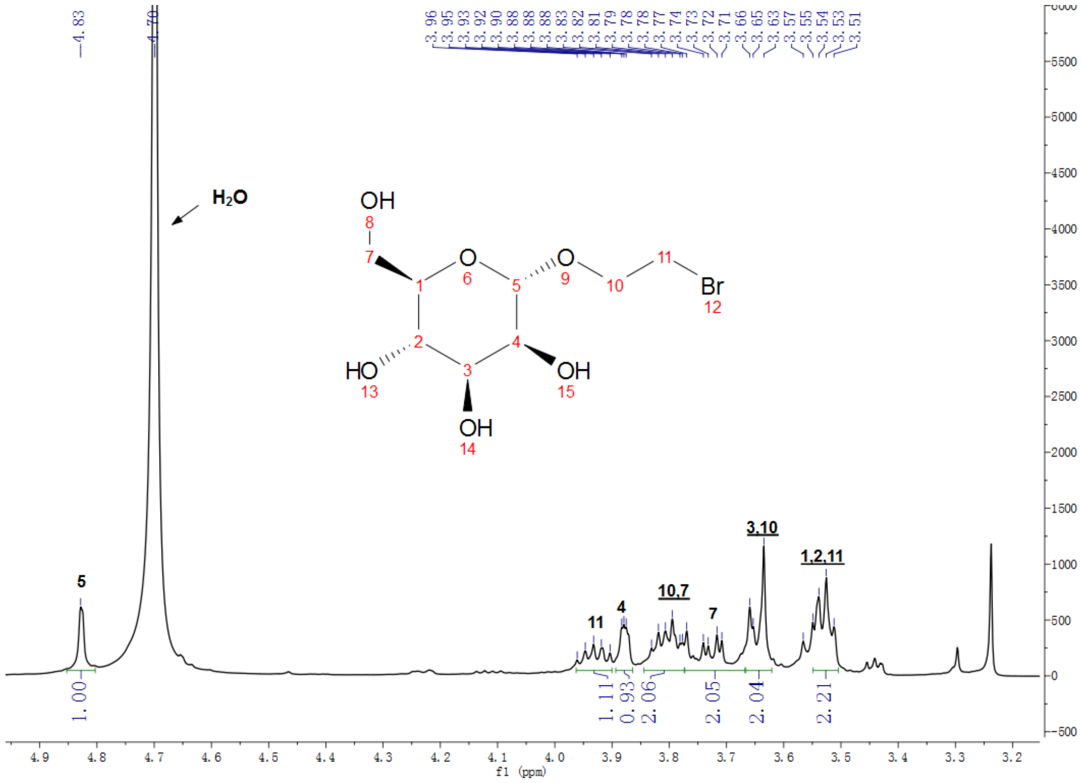

Figure S2. ^{1}H spectrum of **b** recorded in D_2O.

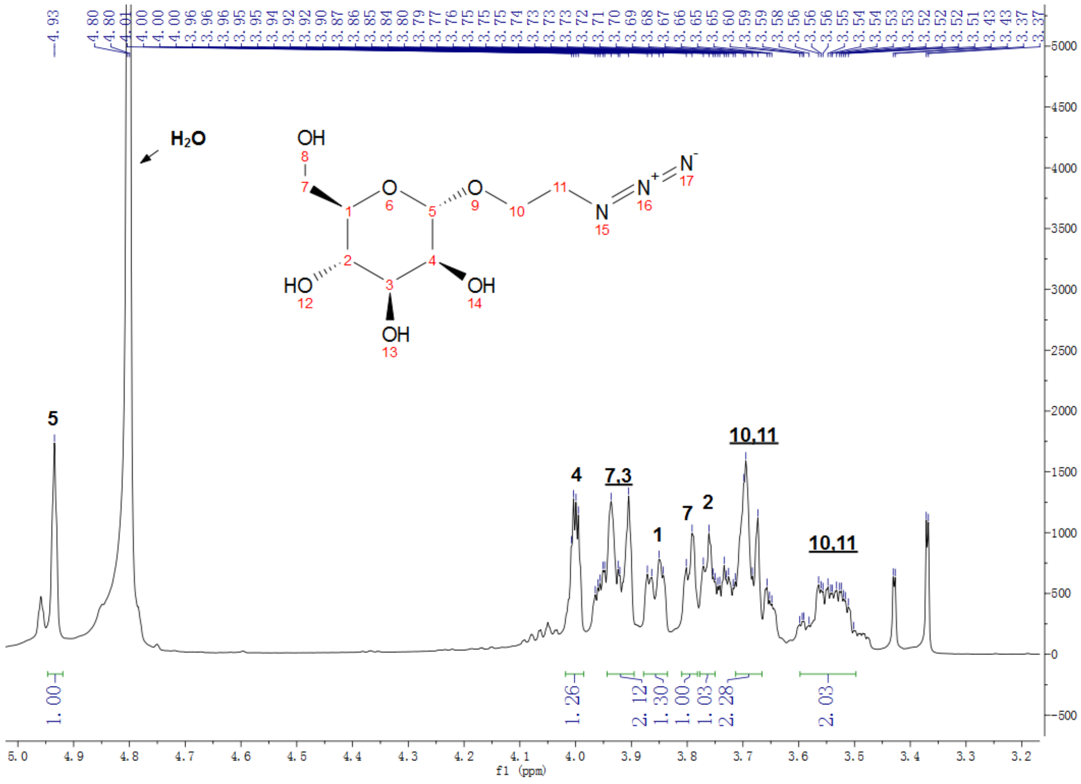

Figure S3. ^{1}H spectrum of **c** recorded in D_2O.

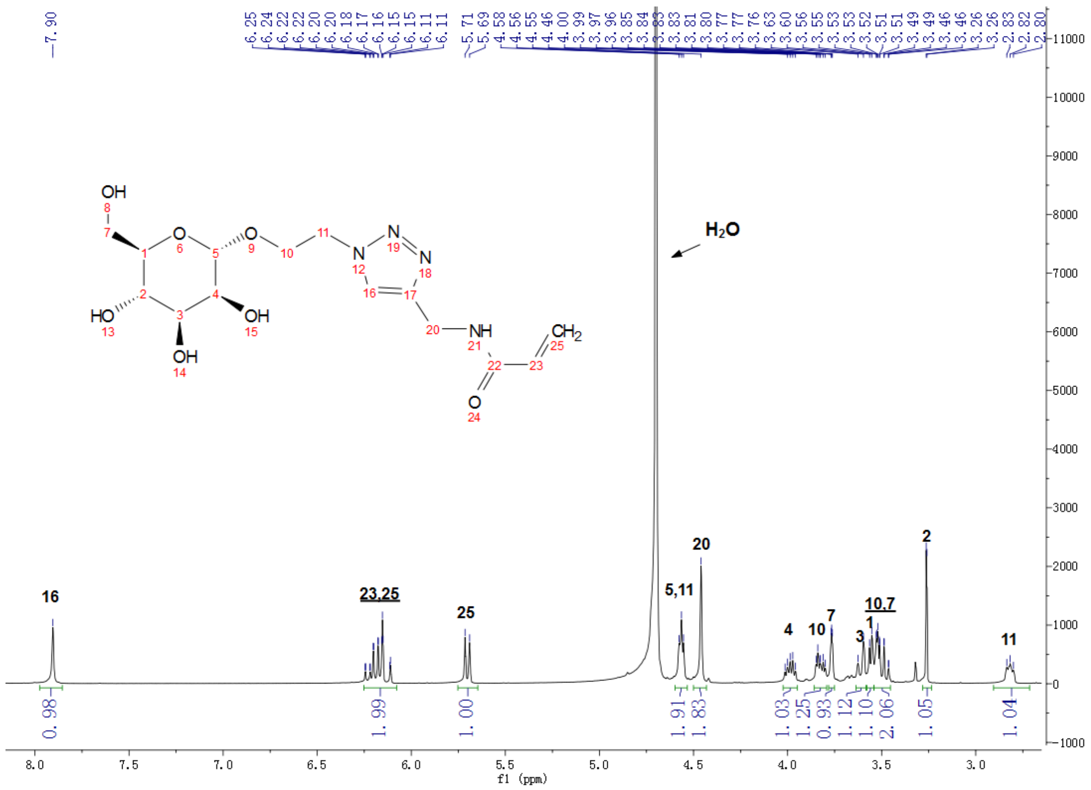

Figure S4. ^1H spectrum of **mannose acrylamide M1** recorded in D_2O.

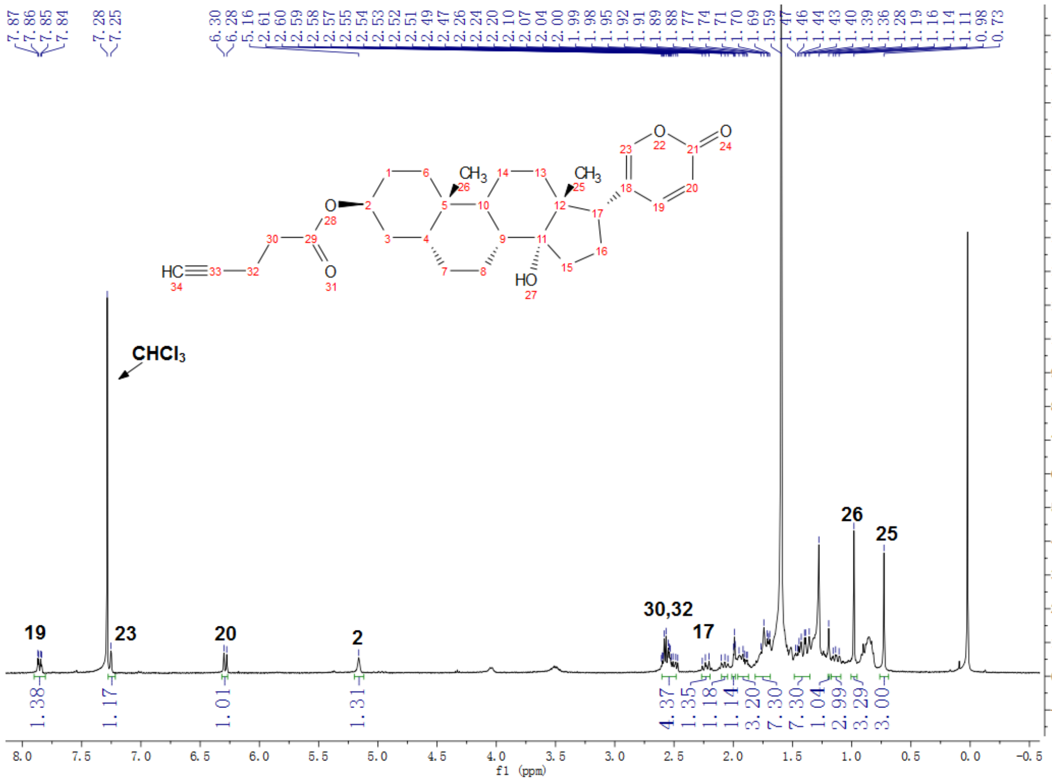

Figure S5. ^1H spectrum of **bufalin ester** recorded in $CDCl_3$.

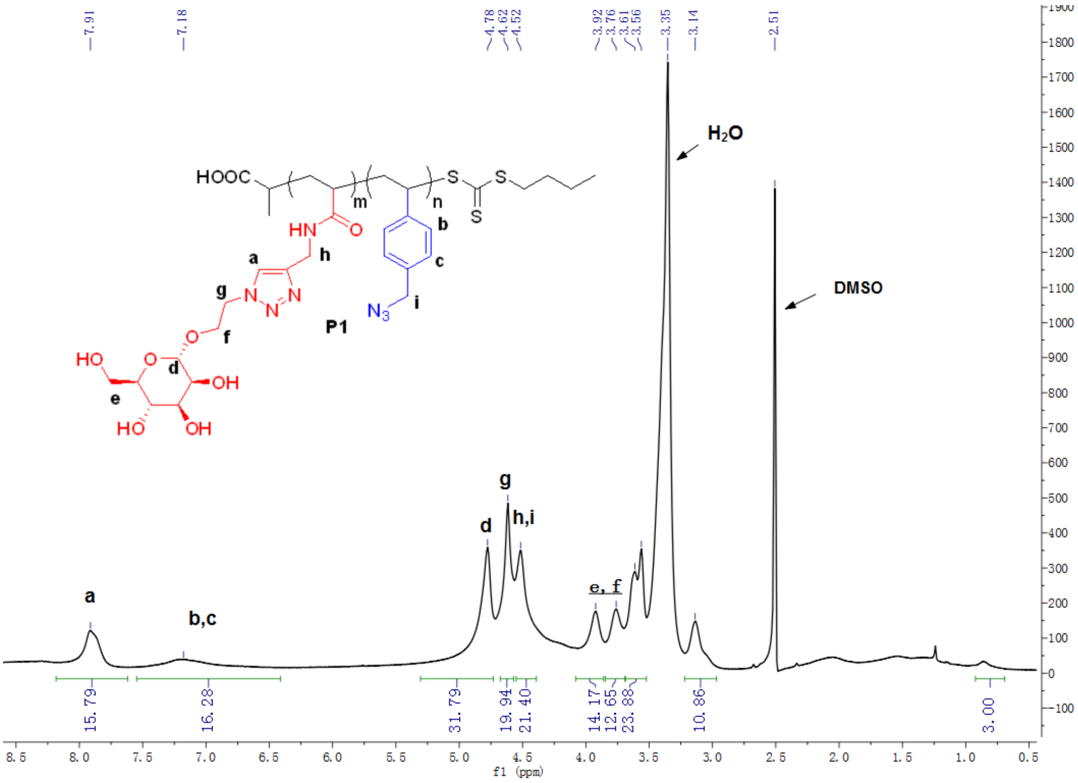

Figure S6. ^1H spectrum of **copolymer P1** recorded in DMSO-d_6.

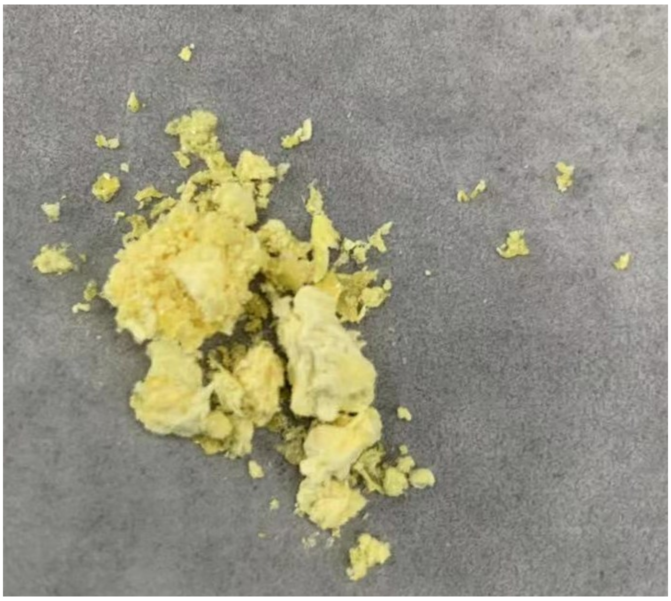

Figure S7. The sample picture of copolymer P1.

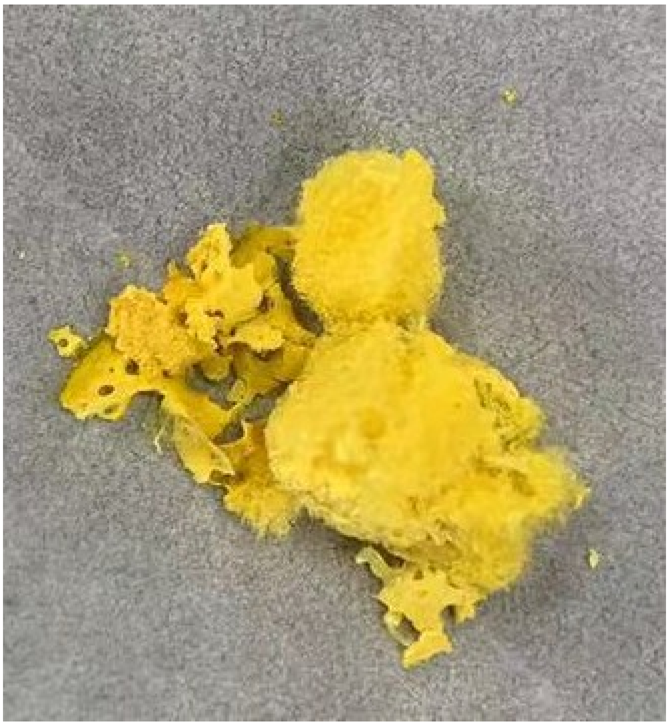

Figure S8. The sample picture of polymer-buf conjugate P2.
